# Supplementary material for: Clustering the protein universe of life using DIAMOND DeepClust
Source: Nat Methods. 2026 Mar 24;23(4):724–7. doi: 10.1038/s41592-026-03030-z (PMC13076203; doi:10.1038/s41592-026-03030-z)
Supplement: Supplementary file 1 — Supplementary Discussions 1–4, Tables 1–4 and Notes. [file 41592_2026_3030_MOESM1_ESM.pdf]

nature methods

Supplementary information https://doi.org/10.1038/s41592-026-03030-z

Clustering the protein universe of life using DIAMOND DeepClust

In the format provided by the authors and unedited

Supplementary Discussion 1

Memory efficiency

The memory optimization feature of DIAMOND allows users to perform large scale searches on their laptops and scale their parallelization efforts seamlessly into an HPC or cloud infrastructure through its distributed memory and parallelization feature. Our aim for DIAMOND DeepClust was therefore to maintain memory efficiency by designing a memory efficient cascaded clustering to allow the clustering of large input datasets on a laptop or massively parallel virtual machines when aiming to scale distributed computing in the cloud. For clustering on HPC systems, the design of the cascaded clustering algorithm as chained rounds of all-vs-all alignments at increasing sensitivity also allows decomposition of this computation into arbitrarily many work packages that can be processed independently on a distributed infrastructure. This process can be automated using the multiprocessing feature introduced in DIAMOND v2. As this technique is not applicable to alignment rounds with linear scaling, we implemented as explicit parallelization of that algorithm.

Supplementary Discussion 2

Cluster extension

Sensitively clustering tens of billions of sequences across the tree of life is a computationally heavy task. However, this procedure has to be performed only once, and the resulting cluster database can be made publicly available **(Data availability)**. To accommodate the future growth of sequenced species within this decade, we provide a cluster extension workflow to allow users to add new query sequences to existing clusters to extend the initial cluster database without the need of re-clustering all sequences together. In particular, new sequences can be searched against the existing representative set using DIAMOND in iterative search mode (option --iterate).

This mode identifies all sequences that can be assigned to an existing cluster. The remaining unaligned sequences can then be clustered independently, and the resulting representatives can be added to the existing clustering.

Supplementary Discussion 3

Projection for Earth BioGenome Era

Clustering the protein universe of our earth's biosphere allows us to quantify and understand the complexities and degrees of divergence when aiming to apply the comparative method across the tree of life and harness insights of relatedness for downstream analyses. To estimate the future applicability of DIAMOND DeepClust, we projected the computational effort of running it on a future dataset that would cover all protein sequences retrieved from the ~1.8 million eukaryotic species expected to be sequenced by the Earth BioGenome Project. To this end, we downloaded the protein sequences of 10,182 eukaryotic assemblies with annotated genes that were available in GenBank as of May 2025. We clustered the protein sequences using DIAMOND DeepClust in very-sensitive mode at an 80% bi-directional coverage cutoff and no identity cutoff. Based on a number of 22,567,737 clusters for this dataset, we project a linear growth of the cluster count as an upper bound estimate, resulting in 3.99 billion clusters for 1.8 million species. The clustering computation is expected to be dominated by all-vs-all alignment in the most sensitive clustering round and can thus be assumed to scale roughly quadratically in the number of clusters. On the basis of this projection assumption and a DIAMOND v2 runtime of 5.48h on a 128-core server for the given computation, we project a computation time of at most 21.9 million CPU hours for processing the full Earth BioGenome dataset with DIAMOND DeepClust. Such a computation is already feasible on HPC systems hosted by the Max Planck Society today and illustrates that the scalability of DIAMOND DeepClust will enable users to learn from the protein sequences of millions of species once they are available. We note that the actual cluster count and run time will likely be much lower due to saturation effects. Using the linear mode of DIAMOND DeepClust

(with multiple spaced seeds for sensitivity) for the same computation resulted in 26,494,861 clusters at a runtime of 3.1h, yielding a projected estimate of 70 thousand CPU hours for processing the full Earth BioGenome dataset, assuming linear scaling.

Supplementary Discussion 4

The observed proportions in our experimental study between singletons and non-singletons are in agreement with previous clustering efforts (Jumper et al., 2021) (Suzek et al., 2007), thereby illustrating that the proportion of singletons does not change much when adding more protein sequences from newly sequenced organisms. To inform ongoing mega-assembly consortia about the proportion of novelty versus assembly artifact we compare the sequence and protein properties of singletons vs non-singletons. First, we filtered out obvious artifacts and poorly annotated sequences by running the repeat masking software tantan (Frith, 2011) with default settings on a random sample of 1,000,000 singletons vs non-singletons to evaluate the proportion of low-complexity sequences among putatively novel proteins. Only 10.5% of proteins were masked over >25% of their range by tantan compared to 9.41% for non-singletons. Next, we compare the length distributions of sequences derived from singleton clusters vs non-singleton clusters, since a well-studied feature of novel proteins is their short average length compared to evolutionarily conserved proteins (Tautz et al., 2011). As a result, we find that singletons are indeed significantly shorter than non-singleton protein sequences with singletons having a median length of 62 amino acids and non-singletons 174 amino acids **(Extended Data fig. 3)**. In addition to sequence length, the entropy in the protein sequence is also known to vary between novel and evolutionarily established proteins (Tautz et al., 2011). Therefore, we calculated the Shannon entropy for all singletons vs non-singletons and observed that singletons indeed show significantly higher entropy values than non-singletons, thereby illustrating the uneven nature of these unique novel proteins (orphan polypeptides) **(Extended Data fig. 3)**. Furthermore, we aligned random

samples of 1,000,000 singletons vs 1,000,000 non-singletons (from clusters containing ≥5 members) against the ECOD database (Cheng et al., 2014) using DIAMOND in ultra-sensitive mode and found that 11,601 sequences (~1.16%) generated alignments with e-value < 0.001, while 180,421 non-singleton sequences (~18%) generated alignments with the same alignment settings. This fact further establishes that the ~1.16 billion singletons we report in this study require further attention to assess their biological relevance. Since these singletons show signatures previously assigned to novel orphan proteins, they encourage further studies. For example, the clustering run used in creating the AlphaFold2 Big Fantastic Database used only 18% of all clusters and constrained the cluster size to at least three elements, while removing 82% of singleton clusters. If, however, further research would reveal that they are the product of poor mega-assembly efforts, our current representation of the protein universe would turn out to be heavily biased by assembly quality, particularly when derived from metagenomic assemblies. Together, our experimental study reveals a possible use case of how the quantification of the protein universe can enable future efforts to test whether more high-quality genome assemblies based on long-read technologies will yield smaller numbers of singleton clusters when joining the tree of life or whether this unique diversity is an intrinsic feature of life itself.

Supplementary Notes

Main benchmark

Annotation

We aligned all 545,815,195 sequences against Pfam-A 33.1 using the pfam_scan.pl script available from InterPro which internally uses HMMER, yielding hits for 420,549,005 sequences. We merged hits in consecutive order along the same sequence against the same Pfam family into a single hit if the model coordinates of the hits were monotonically increasing. We then filtered

the sequences for a minimum of 80% residue-level annotation with Pfam families and determined the domain architecture of each sequence according to the linear order of Pfam annotations along the sequence, resulting in a dataset of 149,824,975 annotated sequences, which we use for the evaluation.

Environment

All tools were compiled natively from source on the host system using GCC 11.4.0. All runs were conducted on a pair of 64-core dedicated virtual cloud nodes with 2 TB RAM and a 2 TB SSD on the HPC Cloud at the Max Planck Computing and Data Facility in Garching. The hypervisors used were dual Intel IceLake-based (Xeon Platinum 8360Y @ 2.4 GHz) compute nodes with a total of 72 cores, 2 TB of RAM and 10 TB SSD storage in RAID 6 configuration. The nodes were dedicated to the project with no other resource allocations to ensure benchmark stability. Network disk storage was provisioned by Ceph block storage based on SATA SSDs in a replicated configuration for the FLSHclust benchmark as it exceeded local disk capacity.

Commands

The commands used to run the tools are available in the respective scripts hosted at https://github.com/bbuchfink/deepclust-data.

DIAMOND v2

We used DIAMOND v2.1.14 available at https://github.com/bbuchfink/diamond. We used the option -DHIT_KEEP_TARGET_ID=ON for compilation, which enables a minor optimization not active by default.

MMseqs2

We used MMseqs2 release 14 available at https://github.com/soedinglab/MMseqs2. To determine the best possible parameter settings, we conducted a set of clustering runs with

different parameters on our subset of 149,824,975 Pfam-annotated sequences from the NR database. We used this subset to make this computation feasible, as clustering the full NR database runs for four weeks using MMseqs2. We assume that the results of this clustering are a sufficient approximation of the full run for the purpose of choosing good parameters. We chose the sensitivity (-s) as the allowed maximum and varied the number of extensions per query (--max-seqs), the bi-directional sequence coverage threshold (-c), the e-value threshold (-e), the use of connected component clustering (--cluster-mode), and the search depth on the graph for connected component clustering (--max-iterations). The following **(supplementary table 1)** lists the parameters of the run and the resulting sensitivity and precision.

Parameters	Sensitivity	Precision
-s 7.5 --max-seqs 1000 -c 0.75 -e 0.1	0.396	0.958
-s 7.5 --max-seqs 1000 -c 0.8 -e 0.001 --cluster-mode 1	0.900	0.540
-s 7.5 --max-seqs 1000 -c 0.8 -e 0.001 --cluster-mode 1 --max-iterations 1	0.505	0.962
-s 7.5 --max-seqs 1000 -c 0.8 -e 0.001 --cluster-mode 1 --max-iterations 2	0.668	0.946
-s 7.5 --max-seqs 500 -c 0.75 -e 0.001 --cluster-mode 1 --max-iterations 2	0.683	0.933
-s 7.5 --max-seqs 500 -c 0.75 -e 0.01 --cluster-mode 1 --max-iterations 2	0.687	0.934
-s 7.5 --max-seqs 500 -c 0.75 -e 0.1 --cluster-mode 1 --max-iterations 2	0.691	0.932
-s 7.5 --max-seqs 500 -c 0.8 -e 0.1 --cluster-mode 1 --max-iterations 2	0.659	0.949
-s 7.5 --max-seqs 500 -c 0.8 -e 0.01 --cluster-mode 1 --max-iterations 2	0.657	0.949
-s 7.5 --max-seqs 500 -c 0.8 -e 0.001 --cluster-mode 1 --max-iterations 2	0.652	0.948
-s 7.5 --max-seqs 500 -c 0.65 -e 0.1 --cluster-mode 1 --max-iterations 1	0.552	0.927

-s 7.5 --max-seqs 500 -c 0.8 -e 0.001 --cluster-mode 1 --max-iterations 3	0.744	0.924
-s 7.5 --max-seqs 500 -c 0.85 -e 0.001 --cluster-mode 1 --max-iterations 3	0.700	0.947
-s 7.5 --max-seqs 500 -c 0.9 -e 0.001 --cluster-mode 1 --max-iterations 3	0.618	0.966
-s 7.5 --max-seqs 500 -c 0.85 -e 0.00001 --cluster-mode 1 --max-iterations 3	0.690	0.950
-s 7.5 --max-seqs 500 -c 0.85 -e 0.01 --cluster-mode 1 --max-iterations 3	0.705	0.945
-s 7.5 --max-seqs 500 -c 0.85 -e 0.1 --cluster-mode 1 --max-iterations 3	0.708	0.946
-s 7.5 --max-seqs 500 -c 0.85 -e 0.0001 --cluster-mode 1 --max-iterations 3	0.694	0.948
-s 7.5 --max-seqs 500 -c 0.85 -e 0.00001 --cluster-mode 1 --max-iterations 4	0.744	0.928
-s 7.5 --max-seqs 500 -c 0.9 -e 0.001 --cluster-mode 1 --max-iterations 4	0.674	0.952
-s 7.5 --max-seqs 500 -c 0.85 -e 0.00001 --cluster-mode 1 --max-iterations 4	0.744	0.928
-s 7.5 --max-seqs 500 -c 0.9 -e 0.001 --cluster-mode 1 --max-iterations 4	0.674	0.952
-s 7.5 --max-seqs 250 -c 0.85 -e 0.001 --cluster-mode 1 --max-iterations 3	0.676	0.954
-s 7.5 --max-seqs 250 -c 0.8 -e 0.001 --cluster-mode 1 --max-iterations 3	0.726	0.934
-s 7.5 --max-seqs 250 -c 0.85 -e 0.1 --cluster-mode 1 --max-iterations 3	0.690	0.952

Supplementary table 1 | MMseqs2 clustering runs with various parameters

We aimed to choose the settings that provided maximal sensitivity at precision of at least 95% and concluded that the highlighted run satisfied this requirement. In particular, we note that connected component clustering (--cluster-mode 1) performed much better than the default mode. A value of 3 for --max-iterations seemed to perform best while a value of 4 (and presumably

higher) reduced precision by too much. We note that increasing the --max-seqs parameter increases sensitivity, run time and disk space usage while reducing precision. For the clustering run of the full NR database, the selected parameters resulted in a disk space use of 510 GB, so it may be possible to slightly improve sensitivity at the expense of longer run times by using a higher value for --max-seqs.

For running MMseqs2 in Linclust mode, we also chose the --cluster-mode 1 --max-iterations 3 parameters along with -c 0.8 --min-seq-id 0 --kmer-per-seq 80 (--kmer-per-seq 80 is the recommended sensitive setting in the Linclust publication). We note that a lower coverage threshold of 80% vs 85% improves sensitivity while still providing precision above 95% for the Linclust mode.

We also attempted to run clustering using profiles as described at https://github.com/soedinglab/MMseqs2/wiki#how-to-cluster-using-profiles. As suggested, we searched our input dataset (the NR database) against the UniRef50 database using this command:

```
mmseqs search nr uniref50 out tmp -a --num-iterations 2 --threads 64
```

This computation expired after running for 3 weeks using 64 cores on an AMD EPYC 7742 system due to a time limit.

FLSHclust

We downloaded FLSHclust at the repository provided in the publication (https://zenodo.org/records/8371343). We used a three-stage cascaded clustering run, chaining the three built-in modes that are recommended in the FLSHclust readme with 0, 6 and 24 hash functions. We used a 20% sequence identity cutoff in all rounds to maximize the performance of the computation. For clustering the full NR database, by advice of the authors, we replaced all ‘X’

residues in the sequence input file by '*' before the second cascaded round, as it would otherwise fail with an error. For the runs on the subsampled databases, we did the same replacement prior to running the tool.

For all tools, we conducted runs with identical parameters on randomly down sampled databases of the NCBI NR, using sample sizes of 270 million, 135 million, 68 million, 27 million and 10 million sequences.

In addition to the runs presented in **(Fig. 1)**, we also conducted a run of DIAMOND DeepClust using a 30% sequence identity threshold and a 90% uni-directional coverage threshold (corresponding to the settings used for the experimental study). Compared to the run using no sequence identity threshold and an 80% uni-directional coverage threshold, this change reduced the sensitivity from 52.3% to 42.5% and increased the precision from 64.9% to 72.3%. The runtime on the benchmark system was 39h 40min.

Memory usage

We report the maximum memory usage (resident set size) of the tools when run for the main benchmark on a server with 64 cores and 2 TB RAM. At least in the case of DIAMOND, these are *not* minimum requirements to run the tool.

**Tool**	**Memory use (GB)**
MMseqs2	608
FLSHclust	1339
DIAMOND DeepClust	1118
DIAMOND DeepClust (linear mode)	982
DIAMOND DeepClust (uni-directional coverage)	1073
MMseqs2/Linclust	843

Supplementary table 2 | Memory usage of tools for the main benchmark.

HMMER benchmark

To benchmark running jackhmmer against 40 billion proteins, we ran it (version 3.4) as single-query searches on 30 query sequences randomly sampled from the UniRef50 database against random samples of the NCBI nr database of increasing size using parameters `--cpu 64 -o /dev/null --noali --F1 0.0005 --F2 0.00005 --F3 0.0000005 --incE 0.0001 -E 0.0001 -N 1` on a virtual server with 64 cores (Xeon Platinum 8360Y @ 2.4 GHz) (**supplementary table 3**). The parameters are corresponding to the default values used by AlphaFold2 according to https://github.com/google-deepmind/alphafold/blob/main/alphafold/data/tools/jackhmmer.py, chosen according to the file because it "speeds up the pipeline at the expensive of sensitivity. They are currently set very low to make querying Mgnify run in a reasonable amount of time.". We recorded actual CPU time using the time utility.

Database size (sequences)	Average CPU time (seconds)
1000000	15.0533
10000000	148.28
27000000	403.597
55000000	840.751
68000000	1034.12
135000000	2056.32
270000000	4075.48

Supplementary table 3 | Average CPU time per query depending on database size

We concluded linear scaling of the runtime in the size of the database and projected a range of 90-400 CPU hours per query for searching against 40 billion proteins.

Experimental study

Since writing this our recommendations to cluster very large datasets have been updated, please refer to https://github.com/bbuchfink/diamond/wiki/How-to-cluster-huge-datasets.

To provide easy access to the 19 billion clustered sequences, we transformed the dataset into an Apache Parquet file accompanied by an index file, also in the Apache Parquet format. This reduces the size of the dataset to 1.7 TB and makes the extraction of specific clusters fast and convenient. The index file contains all clusters with more than 2 members and indicates the Row Group of the dataset file and the corresponding rows, where each cluster can be found. By only loading the specific Row Groups which are smaller than 1 GB into memory, the time and memory usage is small enough to perform the cluster extraction on laptops and desktop computers.

On an external SanDisk Extreme portable SSD with 4TB and 1GB I/O extracting 32,000 clusters containing 1,649,332 sequences with one CPU (Intel Core i5-6500 CPU @ 3.2 GHz) takes 43 minutes, for one cluster with 119 sequences the extraction takes one second.

The Index file, Dataset and a Python script to extract clusters are provided **(Code/Data availability)**.

We first sorted each downloaded FASTA file individually in memory by the length of the sequence in descending order, followed by a global disk-based merge sort on all files using GNU sort, resulting in a combined file of 22,788,215,153 protein sequences. Next, we computed hashes for all sequences which resulted in a deduplicated set of 19,387,935,704 unique sequences.

To limit the use of resources and create checkpoints that could be reverted to in case of an error, we conducted the clustering rounds as an incremental procedure as follows. First, we split the input sequence file into chunks that we processed in sequential steps. Each chunk was first aligned against the current working set of representatives that resulted from the previous steps. Sequences that align against a representative according to the clustering criterion were assigned to its respective cluster. Next, we subjected the remaining sequences that failed to map against an existing representative to all-vs-all alignment at the current round's sensitivity level and determined new representatives using the greedy vertex cover algorithm, which were then added to the working set.

The first round of clustering was performed by incrementally processing 109 chunks of the input file. The command for mapping a chunk to the existing representatives:

```
diamond blastp -q REP_DB -d CHUNK_FILE -c1 -k0 --faster -b300 --lin-
stage1 -f 6 qseqid sseqid corrected_bitscore qstart qend sstart send -
-subject-cover 90 --masking 0 --ext banded-slow --soft-masking tantan
--unaligned-targets UNAL_OUT --approx-id 30 --ignore-warnings
```

For technical reasons, we use the representative database as a query file since this choice significantly accelerates the extension computations in cases where there are sufficiently more targets per query than query sequences per target. Here the --lin-stage1 option manually triggers the Linclust-like logic of only comparing against the longest query sequence for groups of identical seeds. We use the `--unaligned-targets` option to retrieve all targets that did not align against any query, which are then used as input for the following all-vs-all alignment:

```
diamond blastp -q UNALIGNED_SEQS -d UNALIGNED_SEQS -c1 -k0 --faster -
b200 --lin-stage1 -f 6 qseqid sseqid qcovhsp scovhsp
corrected_bitscore --query-or-subject-cover 90 --masking 0 --ext
banded-slow -o SELF_OUT --approx-id 30 --soft-masking tantan --ignore-
warnings
```

We used `diamond greedy-vertex-cover` with the option `--member-cover 90` to compute the clustering based on the alignment output.

We conducted the second round of clustering based on the input file of 4,204,049,109 clusters from the first round, partitioned into 4 chunks, using these commands for mapping and self-alignment:

```
diamond blastp -q CHUNK_FILE -d REP_DB -o OUT --multiprocessing --
parallel-tmpdir TMP_DIR -c1 -b4 --fast --query-cover 90 --approx-id 30
-k1 -f 6 qseqid sseqid corrected_bitscore qstart qend sstart send --
masking 0 --soft-masking tantan --ext banded-slow
```

```
diamond blastp -q UNALIGNED_SEQS -d UNALIGNED_SEQS -o OUT --
multiprocessing --parallel-tmpdir TMP_DIR -c1 -b4 --fast --query-cover
90 --approx-id 30 -k1000 -f 6 qseqid sseqid corrected_bitscore --
masking 0 --soft-masking tantan --ext banded-slow
```

We used `diamond greedy-vertex-cover` with the option `--edge-format triplet` to compute the clustering based on the alignment output. We conducted the third round of clustering based on the input file of 2,223,989,666 clusters from the second round, using this command for self-alignment of the whole dataset:

```
diamond blastp -q INPUT -d INPUT -o OUT --multiprocessing --parallel-
tmpdir TMP_DIR -c1 -b4 --query-cover 90 --approx-id 30 -k1000 -f 6
qseqid sseqid corrected_bitscore qstart qend sstart send --masking 0 -
-soft-masking tantan --ext banded-slow
```

We used `diamond greedy-vertex-cover` with the option `--edge-format triplet` to compute the clustering based on the alignment output. We conducted the fourth round of clustering based on the input file of 1,906,267,323 clusters from the third run, using this command for self-alignment of the whole dataset:

```
diamond blastp -q INPUT -d INPUT -o OUT --multiprocessing --parallel-
tmpdir TMP_DIR --mp-self -c1 -b1 -k0 --more-sensitive --query-or-
subject-cover 90 --approx-id 30 -f 6 qnum snum qcovhsp scovhsp
corrected_bitscore qstart qend sstart send approx_pident evalue --
masking 0 --soft-masking tantan --ext banded-slow --freq-masking
```

We used `diamond greedy-vertex-cover` with the option `--member-cover 90` to compute the clustering based on the alignment output.

Supplementary table 4 | Public protein sequence datasets that were used for our experimental study. For the JGI-IMG database we retrieved only assembled metagenomic sequences which were classified as 'Unrestricted' for the 'JGI Data Utilization Status'.

**Database name**	**Number of sequences**	**Download date/release**	**URL**
IMG Environmental Aquatic Metagenomes	6,232,188,951	March 2022	https://img.jgi.doe.gov/
IMG Environmental Non-Aquatic Metagenomes	6,851,118,226	March 2022	https://img.jgi.doe.gov/
IMG Host-Associated Metagenomes	1,905,657,909	March 2022	https://img.jgi.doe.gov/
IMG Engineered Metagenomes	801,972,930	March 2022	https://img.jgi.doe.gov/
SRC	2,022,891,389	March 2022	http://wwwuser.gwdg.de/~compbiol/plass/current_release/SRC.fasta.gz
MGnify	1,977,479,951	2022_05	https://ebi-metagenomics.github.io/blog/2019/04/10/Protein-database/
metaclust	1,757,323,526	March 2022	https://metaclust.mmseqs.org/2018_06/
NCBI NR	465,406,186	April 2022	https://ftp.ncbi.nlm.nih.gov/blast/db/FASTA/
AGNOSTOS	427,306,945	Ver 5	https://figshare.com/ndownloader/articles/13264769/versions/5
MERC	292,137,902	March 2022	http://wwwuser.gwdg.de/~compbiol/plass/current_release/MERC.fasta.gz
MetaEuk	12,111,301	2019_11	http://wwwuser.gwdg.de/~compbiol/metaeuk/2019_11/
SMAGs	10,207,435	v1	https://www.genoscope.cns.fr/tara/
TOPAZ	8,405,914	v1	https://osf.io/gm564/
GPD	7,581,807	April 2022	ftp.ebi.ac.uk/pub/databases/metagenomics/genome_sets/gut_phage_database
NovelFams	4,587,583	March 2022	https://novelfams.cgmlab.org/
MGV	11,837,198	v1.0	https://portal.nersc.gov/MGV/
**Total**	**22,788,215,153**		

References

1. Jumper, J. *et al*. Highly accurate protein structure prediction with AlphaFold. *Nature* 1–11 (2021).
2. Frith, M. C. A new repeat-masking method enables specific detection of homologous sequences. *Nucleic Acids Res*. **39**, e23 (2011).
3. Tautz, D. & Domazet-Lošo, T. The evolutionary origin of orphan genes. *Nat. Rev. Genet*. **12**, 692–702 (2011).
4. Cheng, H. *et al*. ECOD: an evolutionary classification of protein domains. *PLoS Comput. Biol*. **10**, e1003926 (2014).
